# DrTransformer: heuristic cotranscriptional RNA folding using the nearest neighbor energy model

**DOI:** 10.1093/bioinformatics/btad034

**Published:** 2023-01-19

**Authors:** Stefan Badelt, Ronny Lorenz, Ivo L Hofacker

**Affiliations:** Department of Theoretical Chemistry, University of Vienna, Vienna, Austria; Division of Biology and Biological Engineering, California Institute of Technology, Pasadena, CA, USA; Department of Theoretical Chemistry, University of Vienna, Vienna, Austria; Department of Theoretical Chemistry, University of Vienna, Vienna, Austria; Research Group Bioinformatics and Computational Biology, Faculty of Computer Science, University of Vienna, Vienna, Austria

## Abstract

**Motivation:**

Folding during transcription can have an important influence on the structure and function of RNA molecules, as regions closer to the 5′ end can fold into metastable structures before potentially stronger interactions with the 3′ end become available. Thermodynamic RNA folding models are not suitable to predict structures that result from cotranscriptional folding, as they can only calculate properties of the equilibrium distribution. Other software packages that simulate the kinetic process of RNA folding during transcription exist, but they are mostly applicable for short sequences.

**Results:**

We present a new algorithm that tracks changes to the RNA secondary structure ensemble during transcription. At every transcription step, new representative local minima are identified, a neighborhood relation is defined and transition rates are estimated for kinetic simulations. After every simulation, a part of the ensemble is removed and the remainder is used to search for new representative structures. The presented algorithm is deterministic (up to numeric instabilities of simulations), fast (in comparison with existing methods), and it is capable of folding RNAs much longer than 200 nucleotides.

**Availability and implementation:**

This software is open-source and available at https://github.com/ViennaRNA/drtransformer.

**Supplementary information:**

Supplementary data are available at *Bioinformatics* online.

## 1 Introduction

Most common RNA secondary structure prediction models calculate the thermodynamic minimum free energy (MFE) structure, which assumes that (i) the whole molecule is available, and (ii) the molecule is given sufficient time to fold into the optimal structure. However, cells synthesize RNA molecules in 5′ to 3′ direction via transcription: the RNA polymerase reads the DNA template and appends single nucleotides to the RNA molecule at a rate between 20 and 200 nucleotides per second, although pausing during transcription can last for multiple seconds (Pan and Sosnick, 2006). Typically, the last 14–18 nucleotides are assumed to be caged from the polymerase and prevented from base-pairing, the remaining part of the RNA can fold freely before the full molecule is available.

The expected time for RNA structure formation ranges over many orders of magnitude: from fast hairpin formation and general helix zipping reactions on the order of microseconds (Ma *et al.*, 2006; Pörschke, 1974), branch migration reactions on the order of milliseconds, to other complex rearrangements which may take on the order of seconds or much longer. Accordingly, many experimental findings show that the RNA structures forming during transcription can influence the conformation found at the end of transcription, e.g. folding paths prevent the MFE structure formation (Kramer and Mills, 1981; Xayaphoummine *et al.*, 2007), folding paths speed up MFE structure formation (Heilman-Miller and Woodson, 2003), pausing sites assist the folding of large molecules (Wong *et al.*, 2007), formation of hairpin structures cause the termination of transcription (Roberts, 2019).

*In silico* modeling of cotranscriptional folding is algorithmically challenging, as the ensemble of relevant structures at any particular transcription step can be overwhelming both in terms of computational and visual analysis. Even if only two structures dominate the ensemble in terms of occupancy, many more intermediate structures may have to be included in a model to estimate the dynamics between these two structures. Among existing algorithms are stochastic simulations (Kinfold (Flamm *et al.*, 2000), Kinefold (Xayaphoummine *et al.*, 2005), RNAkinetics (Danilova *et al.*, 2006), CoStochFold (Thanh *et al.*, 2021)], master equation methods [BarMap (Hofacker *et al.*, 2010), theoretical work from Zhao *et al.* (2011)], the deterministic prediction of a single folding trajectory [Kinwalker (Geis *et al.*, 2008)], as well as a recent model to interpret experimental data R2D2 (Yu *et al.*, 2021) and theoretical work on combining stochastic modeling with deterministic helix kinetics (Xu *et al.*, 2022).

The stochastic simulator Kinfold presents the simplest model where changes in secondary structure correspond to elementary moves, i.e. opening and closing of single base-pairs. The main difficulty with this approach, however, is that many simulations are needed to get statistically significant results, leading to an overwhelming amount of data without further post-processing. Typically, both the generation and analysis of Kinfold data are time-consuming and challenging for users.

Here, we present DrTransformer, short for ‘DNA-to-RNA Transformer’: a heuristic for cotranscriptional folding to provide fast approximations of Kinfold simulations. The software is open-source and specifically designed with an easy user interface to make cotranscriptional folding simulations more accessible to the community. We show that the results of DrTransformer compare well to statistically correct sampling of folding trajectories of short sequences. The accuracy of simulations, as well as the limits of sequence length in practice are heavily dependent on structural diversity, cotranscriptional folding traps, and on the chosen parameters. Our concluding runtime estimate uses natural group II intron RNA sequences of 620–781 nucleotides length to demonstrate applicability far beyond the capabilities of any other competing methods besides the overly simplistic model Kinwalker.

## 2 Materials and methods

**Fig. 1.**
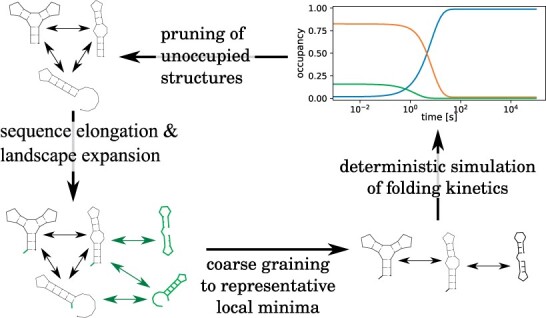
Birds-eye view on the DrTransformer algorithm. For every new nucleotide, the sequence is elongated and the energy landscape is expanded by adding new structures and transition reactions. A coarse graining procedure identifies local minimum conformations to represent the present structure ensemble. A deterministic, kinetic simulation is used to determine how occupancies change in the present ensemble. Before the next nucleotide is transcribed, all unoccupied structures are removed from the landscape

Given a molecule of length *n*, the DrTransformer algorithm proceeds via *n* nucleotide extension cycles (see Fig. 1), which are composed of an *expansion algorithm* where new **candidate structures** are identified, their neighborhood relation in terms of **candidate reactions** is determined and candidate reaction rates are calculated, a *coarse graining algorithm* where the number of candidate structures is reduced, resulting in the so-called **representative structures** and **representative reactions**, a *kinetic simulation* to redistribute occupancies between representative structures until the next nucleotide is transcribed, and a *pruning algorithm* where representative structures with low occupancy are discarded, which yields a set of **parent structures** for the next iteration. Naturally, the algorithm starts with expansion (from the first transcribed nucleotide) and ends with pruning (after the last simulation); we will use this order for discussing the procedures in detail, but start with some formal definitions and background information to level the ground for all sections to come.

### 2.1 Background and notation

We start with the notions of RNA sequence and structure for a molecule of length *n*.

Definition 1*. The* ***sequence***$\sigma_{\left[1,n\right]}$*of an RNA molecule is an ordered list of n nucleotides from 5′ to 3′ end, where* $\sigma_{i}\in \left\{A,C,G,U\right\}$.

Definition 2. *The* ***structure****or* ***secondary structure****x corresponding to RNA sequence σ, is a set of base-pairs (i, j), subject to four conditions: (i) isosteric base-pairs only:* $\left(\sigma_{i},\sigma_{j})\in \{(A,U),(U,A),(C,G),(G,C),(G,U),(U,G)\right\}$*, (ii) every base forms at most one pair: if* (i,j),(i,k)∈x*then j = k, (iii) base-pairs have to be nested: if (i, j) and (p, q) and i < p < j then i < q < j and (iv) hairpins loops contain at least three unpaired nucleotides: if* (i,j)∈x*then* |i−j|>3.

These definitions present a class of nucleic acid structures for which a thermodynamic energy model exists (Turner and Mathews, 2009), and enable the calculation of the MFE conformation as well as the partition function in $O(n^{3})$ time and $O(n^{2})$ space. DrTransformer uses the ViennaRNA package (Lorenz *et al.*, 2011) for secondary structure predictions. Notably, secondary structures as defined here do not include so-called pseudoknots (i.e. conformations containing non-nested base-pairs), and base-triplets (one base engaging in two pairs). Both motifs are present in many interesting RNA structures, but would require a more sophisticated energy evaluation model and substantial adaptations to the presented algorithm.

Definition 3*. An* ***energy landscape***L=(S,M,E)*is a directed, strongly connected graph, where the* ***nodes***x,y∈S*are structures and* ***edges***$m_{x\to y}\in M$*are ‘moves’ corresponding to all possible* ***reactions****(i.e. direct transitions) between structures.* $m_{x⇋y}$*denotes a reversible pair of reactions. Every node* x∈S*has a fitness attribute in form of a thermodynamic* ***free energy***$E_{x}$

Finding the ‘right’ set of structures, the ‘right’ set of reactions and the ‘right’ rates for those reactions in combination with the ‘right’ energy function is the main challenge for this work, and there exist many different approaches to find satisfying and/or practical definitions for those terms in the context of RNA energy landscapes, e.g. Flamm *et al.* (2000, 2002), Kucharik *et al.* (2014) and Entzian *et al.* (2021). We follow the common assumption that the most accurate energy landscape model for secondary structures corresponds to *elementary* base-pair opening and closing steps. This is implemented in Kinfold, a Gillespie-type stochastic simulator for RNA folding, which infers rate constants from the Metropolis (Metropolis *et al.*, 1953) or Kawasaki (Kawasaki, 1966) model. In later sections, we will discuss under which circumstances multiple elementary reactions can be combined into one overall reaction and then use the Arrhenius model to derive reaction rates for transitions that involve multiple base-pair opening and closing steps. The following definitions will be important for the DrTransformer approach to find structures, as well as reactions between those structures with rate constants that are consistent with the energies from the thermodynamic nearest neighbor energy parameters.

Definition 4*. The* ***base-pair distance****d(x, y) between two structures x, y is the cardinality of the symmetric difference between the sets of base-pairs formed by x and y respectively.*

Definition 5*. An* ***elementary path****or* $P_{x⇋y}$*is a sequence of distinct secondary structures, starting in x and ending in y which can be generated via single base-pair opening and closing steps. An elementary path of length* m=d(x,y)*is called* ***direct path****, otherwise it is an* ***indirect path***.

Note that we only use the term direct path in the context of elementary moves, i.e. only single base-pair transitions in the full suboptimal secondary structure ensemble. We will be less strict with the term path, which can refer to an elementary path or a sequence of moves in the landscape L. However, the latter stores the relevant properties of corresponding elementary paths, such as the saddle energy defined below.

Definition 6. *The* ***saddle energy***$E_{x⇋y}$*of a path between two structures* $P_{x⇋y}$*is the maximum free energy on a path:* $E_{x⇋y}=\max_{k\in P_{x⇋y}}E_{k}$.

Definition 7. *A structure x is a* ***local minimum****, if all immediate neighbors have equal or higher free energy. A structure x is a* δ***-minimum****if there exists no structure y with E_y_ < $E_{x}$ reachable by a path with saddle energy* $E_{x⇋y}<\delta$.

While there are known algorithms to identify *δ*-minima considering the full structure ensemble (Entzian and Raden, 2020; Flamm *et al.*, 2002), we take a much faster approach to calculate them only with respect to already observed secondary structures. For example, when we speak of a *δ*-minimum on a direct path, this may or may not correspond to a *δ*-minimum in the full secondary structure ensemble. When we speak of a *δ*-minimum in the landscape L, then this should be understood with respect to all reactions ($m_{x⇋y}\in M$) and their minimal observed saddle energies $E_{x⇋y}$.

Definition 8*. The* ***occupancy****of a structure* $O_{x}\in R^{\left[0,1\right]}$*is a real-valued probability of observing the structure.*

Since the occupancy is a probability, $\sum_{x\in S}O_{x}=1$. We use the term occupancy, to avoid confusion with the thermodynamic equilibrium probability of a structure.

### 2.2 Expansion algorithm

The algorithm maintains a transcript sequence along with a set of secondary structures (x,y∈S) and reactions ($m_{x⇋y}\in M$). The expansion algorithm receives a set of parent structures from the pruning procedure (see Section 2.5), and returns a set of candidate structures and candidate reactions that will be passed on to the coarse graining algorithm (see Section 2.3). Initially, when the first nucleotide is transcribed, the set of parent structures is empty and the expansion algorithm yields only the MFE structure *x* with occupancy *O_x_* = 1. (This yields one unpaired nucleotide, unless the user chooses to start transcription at a larger sequence length.)

After parent structures have been identified from the results of the previous transcription step, the expansion routine adds an unpaired nucleotide to all parent structures. (Readers familiar with the nearest neighbor energy model will note that adding an unpaired base to the end of a structure can change its free energy due to so-called dangling end contributions. As a consequence, also energy barriers involving transitions of parent conformations may change. In order to save computation time, we introduce a small inaccuracy by evaluating the energy of any new structure at the full transcript length, assuming a tail of unpaired nucleotides whenever the transcript is shorter than the full-length molecule.) Those parent structures present the initial set of candidate structures (x,y∈S), and the expansion algorithm proceeds via multiple stages to expand this set of candidate structures and find new candidate reactions ($m_{x⇋y}\in M$). First, a heuristic finds new candidate structures that share base-pairs with parent conformations. Second, a procedure determines a minimal set of candidate reactions ($m_{x⇋y}\in M$)—a so-called **guiding neighborhood**—between candidate structures. This procedure is fast, as it only uses base-pair distances (between each pair of candidate structures) to determine the neighborhood. Third, candidate reactions of the previous transcript step are merged into the set of candidate reactions, and a computationally more demanding routine determines saddle energies and transition rates for all new candidate reactions. Importantly, both the guiding neighborhood construction as well as the algorithm for estimating transition rates are formulated to allow for finding additional candidate structures that may be relevant for kinetic simulations.

#### 2.2.1 Secondary structure search

The core of the secondary structure search procedure is based on the observation that (in the nearest neighbor model) a newly transcribed nucleotide can only interact with bases in the exterior loop, i.e. all nucleotides of the RNA molecule that are not already enclosed by a base-pair, otherwise, a forbidden pseudoknotted structure would be formed. Hence, the structure search is focused on local conformation changes around the exterior loop which are triggered by the newly transcribed base.

**Fig. 2.**
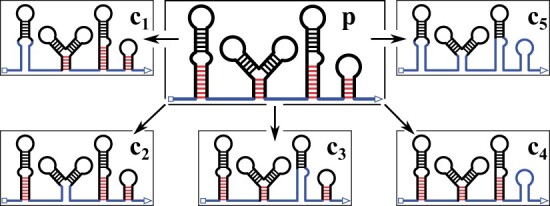
Constraints to generate new candidate structures. The parent conformation *p* has a new unpaired base attached at the 3′ end (triangle) which can pair with any available base in the exterior loop (blue, not enclosed by base-pairs). Fraying helices are shown with red base-pairs (present in p but not in $c_{5}$), black base-pairs are part of enclosed helices (present in all constraints). Each fraying helix is opened separately to produce constraints $c_{1},c_{2},c_{3},c_{4}$, and all fraying helices are opened at once to produce constraint $c_{5}$. The latter allows for rearrangements involving two (or more) competing fraying helices

The parent conformation is translated into multiple constraints that keep all base-pairs and loop regions constant, except for the exterior loop and different combinations of helices adjacent to the exterior loop (the *fraying* helices shown in Fig. 2). If MFE folding subject to these constraints (Lorenz *et al.*, 2016) yields a new (energetically equivalent or better) structure, then it is returned by the structure search algorithm. If fraying liberates less than a minimum number of nucleotides (see option --mfree, Supplementary Section S1) then the next enclosed helix is opened as well. Additionally, the procedure returns the (unconstrained) MFE structure for the current sequence length.

*Intuitions behind the structure search*: Even though we use helix fraying for finding new conformations, the structure where all base-pairs are opened simultaneously is not included into the model, as it is typically not of interest. If a new candidate structure has been determined via constrained folding, then we search for the best direct path saddle energy between conformations afterwards. This may reveal much faster rearrangements that only require partial fraying, or so-called ‘toehold-mediated branch migration reactions’ that does not require any fraying. Unfortunately, the latter reaction type may also be common for conformational rearrangements with pseudoknotted intermediates, but here it can only be found if the toehold forms in the exterior loop.

#### 2.2.2 Guiding neighborhood construction

Guiding neighborhood construction is the first step for finding the initial set of candidate reactions between all candidate structures, and it is independent of known reactions from previous transcription steps. The approach is iterative and proceeds in three steps to construct a so-called ‘guide graph’, whose edges will be the initial candidate reactions for which rates have to be calculated subsequently. First, the current set of candidate structures (nodes) is extended through constrained MFE folding: every structure is used as a constraint to find potentially better structures which are *compatible* with constrained base-pairs. (There is a subtle difference between the constraints used in Sections 2.2.1 and 2.2.2. The former enforce base-pairs and loop regions, the latter only exclude base-pairs that are incompatible with all constrained base-pairs.) Second, base-pair distances are used to find a neighborhood relation between all nodes. Formally, a pair of reversible guide edges $g_{x⇋y}$ is found, if there exists no structure *i*, such that

max{d(x,i),d(i,y)}<d(x,y).

Subsequently, for every node *i*, for every pair of guide edge neighbors *x*, *y*, new shortcut edges $s_{x⇋y}$ are added if

d(x,i)+d(i,y)>d(x,y).

Third, for every guide and shortcut edge, the constrained MFE structure among all structures on direct paths between *x* and *y* is calculated. This is done by allowing only the union of base-pairs of start and end structures, all other base-pairs are forbidden. If this constrained MFE structure does not equal the starting or end structure, it is included into the set of candidate structures. If new structures are found in this third step, then guide neighborhood construction is repeated with the new set of conformations, i.e. current shortcut and guide edges are discarded, otherwise, the algorithm terminates.

*Intuitions behind guide and shortcut edges*: Intuitively, all structures are connected with guide edges, *unless* there exists a structure *in between*. This approach guarantees that every structure is reachable by every other structure in the landscape, and, in the limit of the full set of secondary structures (Wuchty *et al.*, 1999), this approach yields the elementary base-pair opening and closing move set used by statistically accurate stochastic simulators of RNA folding (Flamm *et al.*, 2000). However, the approach has drawbacks if most structures are not explicitly included. As an example, consider the structures x = ..((…)), y = ..(….). and z = (((…))); even though an elementary path for a transition $m_{x⇋z}$ does not have to visit species *y*, only the guide edges $g_{x⇋y}$ and $g_{y⇋z}$ are found. Connecting ‘neighbors of neighbors’ whenever there exists a shorter path is a comparatively simple solution that fixes the problem stated above, while maintaining correctness in the limit of the full set of suboptimal structures where no shortcut edges would be added.

#### 2.2.3 Estimating transition rates between structures

The last part of expansion is to analyze folding paths and estimate reasonable transition rates. It is worth emphasizing that the following algorithm assumes that the most important candidate structures are already known, but additional candidate structures must be included whenever the probability of a transition cannot be described by a single rate constant. As the following procedure to determine saddle energies is time-consuming, we keep a cache of candidate reactions from the previous transcript step and only process newly discovered edges from the guide graph.

**Fig. 3.**
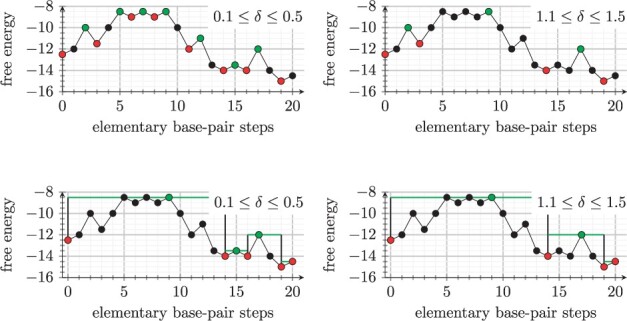
Results of the path flooding procedure in terms of direct transitions between *δ*-minima. All images show the free energy change along a direct path with 20 elementary steps. Every point corresponds to a structure, *δ*-minima are highlighted in red and saddle points are highlighted in green. (Top row) *δ*-minima and saddle points as identified by the flooding procedure with 0.1≤δ≤0.5 and 1.1≤δ≤1.5, respectively. The coloring assumes that nodes at the same energy level are processed from left to right by the flooding procedure. (Bottom row) Direct transitions included as candidate reactions for the given *δ*. All *δ*-minima with energy lower than starting and end conformations are explicitly included in the set of candidate secondary structures, the saddle energy (green line) is used to estimate transition rates between candidate structures

For every guide and shortcut edge, a direct path $P_{x⇋y}$ is generated using the findpath heuristic (Flamm *et al.*, 2001); findpath searches for a path with minimal saddle energy among all direct paths in the full secondary structure ensemble. The path presents a one-dimensional energy landscape where *δ*-minima and saddle energies (see Section 2.1) can be determined using a flooding algorithm (see Fig. 3). Only if the path has no *δ*-minimum k≠x≠y with $E_{k}\leq \max(E_{x},E_{y})$, a direct transition has been found. In that case, two valid transition edges $m_{x⇋y}$ are added to the set of edges M and the saddle energy $E_{x⇋y}$ is used to calculate a reaction rate constant using the Arrhenius model

(1) $$k_{x\to y}=k_{0}e^{-\frac{E_{x}-E_{x⇋y}}{RT}}$$

where the pre-exponential factor *k*_0_ is a rate constant to map simulated time scales to the wall-clock time observed in experiments, *R* is the gas constant and *T* is the temperature. Otherwise, if new *δ*-minima k≠x≠y with $E_{k}\leq \max(E_{x},E_{y})$ are found, then those structures are included into the set of candidate structures and new saddle energies are calculated for the subpaths.

*Intuitions behind the transition rate model*: In a full suboptimal structure space, the guiding neighborhood in combination with this rate model yields elementary base-pair moves with Metropolis rates. In a sparse structure space, where elementary paths determine the transition rate, we assume that if a *δ*-minimum *k* has energy $E_{k}>\max(E_{x},E_{y})$, then it is short-lived, and the overall reaction rate between structures *x* and *y* is dominated by the saddle energy $E_{x⇋y}$. Otherwise, if *k* has energy $E_{k}\leq \max(E_{x},E_{y})$, then this assumption cannot be made and structure *k* has to be included into the set of candidate structures.

### 2.3 Coarse graining algorithm

Coarse graining uses a separation of timescales to reduce the number of candidate structures to fewer, distinct, *δ*-minimum conformations, so-called representative structures. The presented algorithm identifies representative reactions between the remaining set of representative structures and approximates their rates from minimal saddle energies among all previous candidate reactions. Specifically, all reactions between two structures that are separated by a low energy barrier are assumed to be fast (effectively instantaneous); all other reactions are used for simulation.

#### 2.3.1 Top-down coarse graining

**Fig. 4.**
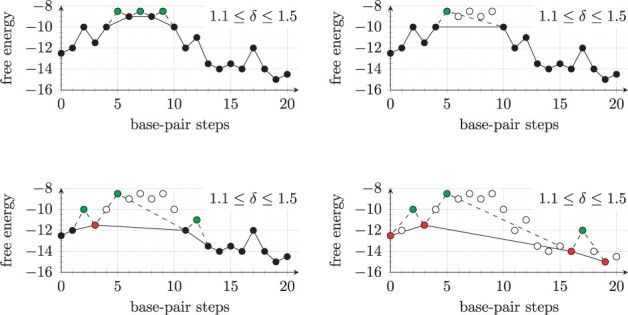
The top-down coarse graining procedure illustrated using an hypothetical direct path example. Four characteristic steps are shown. Top left, the three structures with highest energy have been processed. They are removed from the set of structures, but their energy still determines the transition rate between neighbors. Top right, two more structures have been removed, only one transition is left and the energy barrier is given by a saddle point structure (green, connected with dashed lines). Bottom left, the first *δ*-minimum has been identified (red, connected to two saddle point structures). This structure cannot be removed from the system because it has no outgoing fast reaction. Bottom right, the final coarse graining consists of four *δ*-minimum conformations and three reversible direct transitions where the rate is calculated from the energy of saddle points

Figure 4 illustrates the algorithm using a simple 1D toy energy landscape, the generalization to high dimensions will be described below. Note that this is the same landscape as in Figure 3, where we describe a flooding algorithm to identify *δ*-minima on direct paths. The top-down coarse graining algorithm processes a list of structures sorted from high energy to low energy (the order of structures at the same energy level is irrelevant). For every conformation *k*, take the set of outgoing reactions that yield a conformation with lower or equal energy. If any of these reactions has saddle energy $E_{k⇋x}<E_{k}+\delta$ (i.e. it is *fast*), then *k* is a *transient* structure which will be ignored, otherwise it is a *δ*-minimum and added to the set of representative structures.

If *k* is a transient structure, then its occupancy is divided among the neighbors *x* where $E_{k⇋x}$ is minimal. Note that all higher energy neighbors must be *δ*-minima, otherwise they would have been removed from the landscape (a *δ*-minimum cannot be reached by a fast reaction from a structure with lower energy). Every neighbor *x* of *k* which is reachable by a fast reaction is connected with all other neighbors *y* of *k*. In other words, if two neighbors *x*, *y* are both only reachable by slow reactions, then they are not connected.

*Limitations of top-down coarse graining*: Notably, this algorithm does not account for the entropy of representative structures, i.e. the partition function of all structures represented by *δ*-minima. Adapting the algorithm to incorporate entropy is not straight-forward, as both the free energies of *δ*-minima as well as transition rates have to be adjusted accordingly.

### 2.4 Kinetic simulation

The rates of representative reactions are written into a rate matrix

(2) $$R_{ij}=\{\begin{array}{c} R_{ii}=-\sum_{j}k_{i\to j}\\ R_{ij}=k_{j\to i} \end{array}$$

where every row *i* corresponds to the constants of the linear equation $\frac{di}{dt}=0$ that must be satisfied at thermodynamic equilibrium t→∞. In combination with the vector of initial occupancies ${\vec{p}}_{0}$, any vector ${\vec{p}}_{t}$ can be derived using

(3) $${\vec{p}}_{t}=e^{Rt}{\vec{p}}_{0}\;.$$

where the matrix exponential $e^{Rt}$ can be calculated efficiently in various ways for less than order 10 000 species (Moler and Van Loan, 2003). The two specific approaches described below have been implemented previously in the program treekin (Wolfinger *et al.*, 2004), but DrTransformer provides a standalone implementation of this process using the well-known Python libraries numpy (Harris *et al.*, 2020) and scipy (Virtanen *et al.*, 2020). First, $e^{Rt}$ can be calculated directly using the Pade approximation which is remarkably stable against numeric instabilities, but comparatively slow when calculating many different time points. The more efficient solution decomposes the matrix into a matrix of eigenvectors *S* and a diagonal matrix of eigenvalues Λ to solve the equation $e^{Rt}=Se^{\Lambda t}S^{-1}$. In this solution, matrix decomposition is the time-consuming part, but $e^{\Lambda t}$ for every time-point *t* can be calculated in linear time. In order to counteract numeric instabilities and avoid complex solutions, we first use the detailed balance property of our system ($P_{i}k_{ij}=P_{j}k_{ji}$) to derive the equilibrium distribution vector ${\vec{p}}_{\infty }$, and to derive a symmetric matrix $U=\Omega^{-1}R\Omega$, where Ω is a diagonal matrix with elements $\Omega_{ii}=\sqrt{{\vec{p}}_{\infty }[i]}$. Now the symmetric matrix is decomposed into eigenvectors and eigenvalues $U=S\Lambda S^{-1}$, and Equation (3) can then be rewritten as

(4) $${\vec{p}}_{t}=\Omega Se^{\Lambda t}S^{-1}\Omega^{-1}{\vec{p}}_{0}$$

where ΩS and $S^{-1}\Omega^{-1}{\vec{p}}_{0}$ are constant with respect to changes over time *t*.

Every simulation has a linear regime $\left[t_{0},t_{1}\right]$ and a logarithmic regime $\left(t_{1},t_{8}\right]$. The linear regime simulates folding kinetics for the time interval of nucleotide extension, e.g. $t_{1}=0.04$ seconds at a transcription rate of 25 nucleotides per second. The logarithmic regime is primarily used to simulate folding kinetics after transcription, which can be a much longer time period than cotranscriptional folding itself. During transcription, the logarithmic regime is used to ‘look ahead’ until the end of transcription, i.e. $t_{8}=\sum_{k=l+1}^{L}t_{1}^{k}$ where *l* is the length of the current transcript, *L* is the length of the full sequence and $t_{1}^{k}$ is *t*_1_ at length *k*. This look-ahead simulation is used to find structures which should be exempt from pruning (which will be described in Section 2.5), because they reach a high occupancy at the time scale of transcription. After transcription, *t*_8_ is set to the final post-transcriptional simulation time.

### 2.5 Pruning algorithm

Pruning connects simulation results (Section 2.4) with the next round of expansion (Section 2.2) and the algorithm is partly intertwined with of both of those processes. The general idea is that occupancies of representative structures change, and low-occupied representative structures are discarded in order to keep the system computationally tractable.

We say that any representative structure whose occupancy remained under a threshold *o* during the look-ahead simulation described in Section 2.4 is a *prunable* structure. All prunable structures are sorted from lowest to highest occupancy, and removed from representative structures as long as their combined occupancy remains below the threshold. The remaining set of representative conformations forms the set of parent structures for the next iteration of the algorithm. Hence, it is guaranteed that parent structures x∈S had a combined occupancy $\sum_{x\in S}O_{x}\geq 1-o$.

When representative structures are removed, their occupancy is distributed evenly among all remaining, neighboring, representative structures in the landscape L, or recursively, among neighbors of neighbors if all of the neighboring representative structures are also removed. However, only structures that are not re-discovered as candidate structures during expansion have their occupancy actually distributed among neighboring conformations. We provide an option to set the parameter *o* that specifies the maximum amount of density that can be discarded from the current occupancy distribution.

## 3 Results

**Fig. 5.**
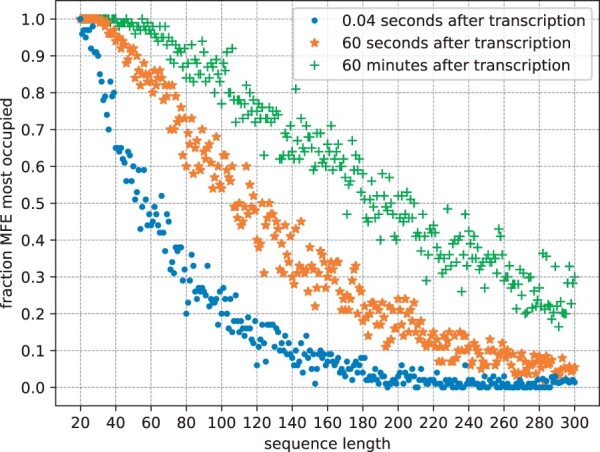
How often is the most occupied structure after transcription the MFE structure? We compare 0.04 s after transcription (the time at which the next nucleotide would be attached at a transcription rate of 25 nt s^−1^), 60 s after transcription and 60 min after transcription. DrTransformer simulations suggest a rapid decline, e.g. <50% of 60 nt sequences are in their MFE conformation at the end of transcription, and <50% of 200 nt sequences are in their MFE conformation one hour after transcription. Each data point is the average of DrTransformer simulations for 100 random sequences of that length

Cotranscriptional folding can have a strong influence on the predicted structure even for comparatively short sequences. The data from Figure 5 suggests that <50% of random sequences with 120 nucleotides are at equilibrium 60 s after transcription.

Generally, many parameters can influence DrTransformer simulations in subtle ways to drastically change the conformations found at the end of transcription. For example, the coarse graining strength reduces the number of representative structures, the occupancy cutoff parameter may discard conformations before they become relevant for expanding the network. Obviously, the kinetic simulation time per nucleotide can have a particularly strong influence on cotranscriptional simulation results. We provide a summary of default parameters along with a brief discussion on their effects in Supplementary Section S1. In Supplementary Section S2, we analyze the secondary structure prediction capabilities of DrTransformer on tRNA structures compared to the thermodynamic model and we show that parameter settings have little influence on the overall quality of predictions.

As DrTransformer is a heuristic approach to cotranscriptional folding on an energy landscape with elementary base-pair transitions, here we focus on the differences of cotranscriptional ensemble predictions when comparing DrTransformer with the underlying ground truth folding model implemented in the program Kinfold. We illustrate the differences using an experimentally verified system and provide an additional analysis using random sequences to assess the diversity of cotranscriptional ensembles from DrTransformer and Kinfold and their correspondence to the equilibrium distribution in Supplementary Section S3.

### 3.1 Estimation of a suitable *k*_0_ parameter

For didactic purposes, we distinguish two parameters that influence the simulation time per nucleotide: The *k*_0_ rate constant of the Arrhenius-type rate model used by DrTransformer [see Equation (1)] translates arbitrary time units given by free energy differences to wall-clock time in seconds, and the extension time specifies how many seconds to simulate per nucleotide. (It is obvious that those parameters are dependent, e.g. doubling both parameters yields the same simulation time per nucleotide, but it is more natural to fix *k*_0_ to a commonly accepted value, and then vary the transcription rate in terms of nucleotides per second.)

In past contributions analyzing cotranscriptional folding using Kinfold (Helmling *et al.*, 2017) and BarMap (Badelt *et al.*, 2015), 4000 arbitrary simulation time units per nucleotide were used in combination with the Metropolis rate model. As DrTransformer uses the same energy model and is an approximation to Kinfold simulations, we expect that the simulation time per nucleotide should be on the same order of magnitude. In particular, we expect that DrTransformer can produce similar results to Kinfold and that differences in simulation results may be compensated by minor adaptations to the *k*_0_ parameter.

**Fig. 6.**
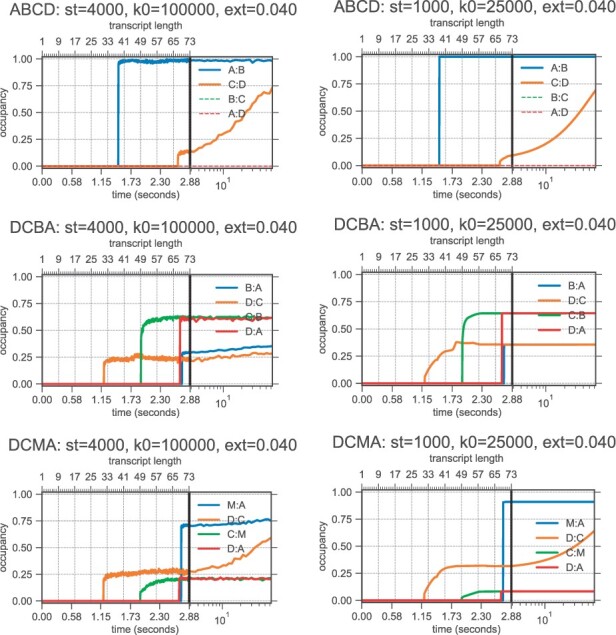
An example for adjusting the rate parameter *k*_0_ (which relates free energy differences to wall-clock time) to improve correspondence between DrTransformer and data. Here, we aim to match ‘ground truth’ Kinfold simulations using sequences ’ABCD’, ’DCBA’, ’DCMA’ from Xayaphoummine *et al.* (2007). (Note that this is much more difficult than fitting to the available experimental data for the structure distribution at the end of transcription.) The trajectories show fractions of structures that form the respective helices. All simulations use extension time ext** **=** **0.040 s nt^−1^, which corresponds to a transcription rate of 25 nt s^−1^. (First column) Kinfold simulations using $k_{0}=10^{5}$. (Second column) DrTransformer simulations using $k_{0}=2.5\cdot 10^{4}$. Varying *k*_0_ effectively changes the simulation time in arbitrary units per nucleotide (termed **st** in plot headers). For further variation of simulation parameters, see Supplementary Section S2

The simulations in Figure 6 correspond to three different RNA molecules from an experimental study (Xayaphoummine *et al.*, 2007) that illustrates how helix competitions determine the structure formed at the end of transcription. Briefly, two sequences are composed of the same palindromic subsequences (A, B, C, D) in forward and reverse order (‘ABCD’ and ‘DCBA’); the third sequence (‘DCMA’) has a point mutation which changes B to M. The experiment demonstrates how the order of helix formation determines which structures are formed at the end of transcription, an effect that cannot be observed with a thermodynamic equilibrium prediction, because the free energies of, e.g. the helices A:B and B:A are the same due to their palindromic subsequences.

For Kinfold simulations, we use the Metropolis rate model with parameter $k_{0}=10^{5}$s^−1^, in combination with a transcription rate of 25 nt s^−1^. The Kinfold simulations explain experimental findings regarding the ensemble at the end of transcription (see Figure 6, first column): ABCD folds almost exclusively into the MFE structure which forms only helices A:B and C:D, while the reverse sequence DCBA is cotranscriptionally trapped to form predominantly helices D:A and C:B. The single-base-mutation in DCMA decreases the effect of the cotranscriptional folding trap and helices D:C and M:A are favored at the end of transcription. Figure 6, second column, shows the cotranscriptional folding simulations of all three sequences using DrTransformer. Varying the simulation time per nucleotide (here by changing *k*_0_) yields a range of different simulation results. The simulations using $k_{0}=2.5\cdot 10^{4}$s^−1^ come close to Kinfold predictions, suggesting that DrTransformer simulations for this example are approximately a factor 4 faster than Kinfold simulations. We show an additional variation of simulation parameters in Supplementary Figures S3–S6, with a discussion on how they relate to experimental findings. (Interestingly, the ratio of structures forming D:C and B:A versus structures forming D:A, C:B in the *DCBA* molecule is dependent on the transcription rate, an effect that can be observed from DrTransformer as well as Kinfold simulations, but—to our knowledge—has not been investigated experimentally.)

### 3.2 Performance analysis of DrTransformer

**Fig. 7.**
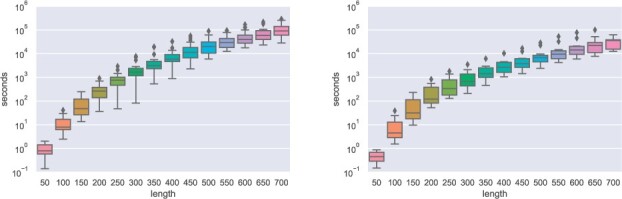
Runtime comparison of DrTransformer for random sequences up to 700 nt length and natural group II intron sequences (620–781 nucleotides length). Relevant non-default transcription parameters: --o-prune = 0.1

Figure 7 shows runtime of DrTransformer as a function of sequence length. We compare two different datasets:

- 20 random sequences up to a length of 700 nucleotides; and
- 11 group II intron sequences (620 to 781 nucleotides length).

The runtime can vary by more than an order of magnitude for different sequences of the same length. This is expected, as runtime depends on properties of the energy landscape, which are not known prior to the simulation. Also, the runtime for random sequences appears worse than for natural group II intron sequences. This may suggest that random sequences have a more diverse energy landscape and thus take longer to simulate. To produce the random sequence data plot, random 900 nt sequences were generated and given up to 7 days ($\approx 6*10^{5}$ s) on a single core for DrTransformer simulations, respectively. Two out of 20 simulations did not terminate in time, one of them reached 896 nt length, the other only 743 nt. Hence, plots show the runtime up to 700 nucleotides. All group II intron sequences were simulated until the last nucleotide.

## 4 Discussion

We have presented a heuristic for cotranscriptional folding that is applicable to both short and long RNA molecules. The Arrhenius-type rate model used by DrTransformer [see Equation (1)] is a generalized formulation of the Metropolis model, as it yields the same rates for single base-pair moves, but also allows for an estimation of transition rates that involve multiple base-pair insertions/deletions at once. While our algorithm compresses elementary paths into single steps (which can speed up simulations) there may exist saddle structures with lower energy than those found on direct paths, in which case DrTransformer simulations would be slower than the corresponding Kinfold approach. However, we have shown that, in practice, DrTransformer is able to qualitatively reproduce simulation results of the much more computationally expensive ground truth Kinfold approach via slight adjustments to the *k*_0_ parameter.

*Usage notes*: As mentioned for the sequences in Figure 6, folding behavior can depend on the *k*_0_ parameter and the transcription rate—the latter is often not precisely known. Thus, it is recommended to vary the time per nucleotide to observe different types of structures at the end of transcription. If experimental results are known, the user can adjust the *k*_0_ parameter to plot results with a matching transcription rate. DrTransformer also provides options for pause sites at specific nucleotides, which in principle could be used to see how stochastic variations of the transcription rate (at each nucleotide) influence folding. Apart from options that influence the DrTransformer heuristic directly, DrTransformer provides an interface to all relevant ViennaRNA package energy model parameters such as temperature and alternative nearest neighbor parameters.

*Future work*: The presumed ground truth Metropolis model for RNA folding is limited, as it depends on a single parameter to adjust how differences in free energy correspond to wall-clock time. It is also not clear whether intermediate steps during a helix-zipping reaction automatically correspond to well-defined Markovian minima as presumed by Kinfold. DrTransformer provides a basis to test new rate models in combination with experimental data on longer RNA molecules. For example, Zolaktaf *et al.* (2017) use an Arrhenius-type model for DNA folding kinetics with additional parameters for secondary structure context.

While DrTransformer provides parameters for simulations of long RNA molecules, more work is needed to determine parameters for which such predictions match experimental results. Many longer RNA molecules use specific types of pseudoknots to assist the folding into the target structure. Cotranscriptional folding of long RNAs may be especially interesting in the field of RNA origami (Geary *et al.*, 2014), which typically relies on such types of interactions. While it seems difficult to improve DrTransformer predictions by allowing pseudoknots in general, a stepwise inclusion of certain classes in combination with a well-described kinetic model is definitely an exciting direction.

## 5 Conclusion

DrTransformer presents a deterministic approach for cotranscriptional folding using heuristic energy landscapes for every transcription step. The program can be viewed as a hybrid approach between BarMap, a deterministic simulation on a priori coarse-grained landscapes (Hofacker *et al.*, 2010), and Kinwalker, a greedy algorithm to get the most probable trajectory (Geis *et al.*, 2008). In practice, DrTransformer can produce similar results to Kinfold using much less computation time, which allows users to scan over multiple possible transcription parameters quickly.

Immediate use cases for DrTransformer are the identification of cotranscriptional folding traps, and the analysis of cotranscriptional pausing sites with respect to secondary structure formation. More subtle analyses could involve the identification of sequence regions where certain transcription parameters are essential for correct folding, or where experimentally observed folds cannot be realized. The latter would suggest crucial interactions with unknown molecules that assist for correct folding. For example, it has recently been found that transient, non-native structures kick-start ribosome assembly [reviewed in Rodgers and Woodson (2021)]. Finally, DrTransformer opens a variety of possibilities for sequence design, as the evaluation on whether an intended folding path is cotranscriptionally favorable is now much faster than using previous methods. For example, DrTransformer may be able to identify (and avoid) cotranscriptionally formed transcription termination motifs, which would greatly assist the design of large RNA molecules.

## Supplementary Material

btad034_Supplementary_Data

## Data Availability

The software and example sequences used for the paper are available at https://github.com/ViennaRNA/drtransformer.
