# Supplementary material for: DrTransformer: heuristic cotranscriptional RNA folding using the nearest neighbor energy model
Source: Bioinformatics. 2023 Jan 19;39(1):btad034. doi: 10.1093/bioinformatics/btad034 (PMC9889959; doi:10.1093/bioinformatics/btad034)
Supplement: btad034_Supplementary_Data [file btad034_supplementary_data.pdf]

`DrTransformer`: Heuristic cotranscriptional RNA folding using the nearest neighbor energy model. - Supplementary Material

Stefan Badelt [1,2,*], Ronny Lorenz [1], and Ivo L. Hofacker [1,3]

January 11, 2023

[1]Department of Theoretical Chemistry, University of Vienna, Austria.
[2]Division of Biology and Biological Engineering, California Institute of Technology, Pasadena, CA, USA
[3]Research Group Bioinformatics and Computational Biology, Faculty of Computer Science, University of Vienna, Austria

[*]To whom correspondence should be addressed

Option	Default	Explanations
--t-ext	0.04	The simulation time in $\mathrm{s\,nt^{-1}}$ during transcription.
--t-end	60	The simulation time in s after transcription.
--t-lin	10	Evenly space output --t-lin times on the linear time scale.
--t-log	30	Evenly space output --t-log times on the logarithmic time scale.
--mfree	6	The minimum number of freed bases during helix fraying.
--fpwm	4	The findpath search width multiplier.
--k0	10^5	The rate constant for the Arrhenius-type folding kinetics model.
--t-fast	0.004	Coarse graining threshold to determine instantaneous reactions.
--o-prune	0.05	Occupancy threshold for determining parent structures after simulations.
--delth	10	Cache previous structures and reactions for --delth transcription steps.

Table 1: Summary of algorithmic parameters for DrTransformer

1 DrTransformer algorithm parameters

Table 1 shows the default parameters of DrTransformer v.1.0. Unless explicitly stated otherwise, these parameters were used for analysis of systems in the main text and supplemental material. The options --t-ext, --t-end, --t-lin, and --t-log set the simulation time per nucleotide as well as the number of returned output time points. The default --t-ext = $0.04\,\mathrm{s\,nt^{-1}}$ corresponds to a transcription rate of $25\,\mathrm{nt\,s^{-1}}$. For reference, transcription rates range between $20 - 200\,\mathrm{nt\,s^{-1}}$. (DrTransformer also provides an option to set pause sites; see DrTransformer --help). The default parameter --t-end = 60 s should be adjusted to however long the user wants to track folding kinetics after transcription. The only limitation is that --t-end $\geq$ --t-ext. Because transcription is a relatively fast process compared to the lifespan of an RNA molecule, we use a linear time scale for reporting the change of occupancies during transcription and a logarithmic time scale for reporting the change of occupancies after transcription. The options --t-lin and --t-log adjust how many time points should be reported for either of those regimes. Smaller values produce less data for further processing.

The parameter --mfree sets the minimum number of freed bases during helix fraying. For example, six could correspond to two base-pairs and an interior loop with two unpaired bases. We do not recommend a smaller value than six, e.g., if --mfree ≤ 2, then a single lonely base-pair (Bompfünewerer *et al.*, 2008) in the exterior loop prevents the generation of further constraints. However, it may make sense to increase the parameter to find structures that have less similarity with parent conformations. When using an excessively large value, all structures that are enclosed by a base-pair in the exterior loop must open completely, one-by-one.

The parameter --fpwm is a multiplier to set the search width w for finding direct path saddle energies using findpath (Flamm *et al.*, 2001) as $w = d(x, y) \cdot$ --fpwm. Larger values generally yield more accurate results but increase runtime as findpath has a complexity of $O(d(x, y)^2 w)$.

The parameter --k0 sets the rate constant k_0 from the Arrhenius model (see Eq. 1, main text). In the Arrhenius model, k_0 describes both the fastest possible reaction rate in a system, which is on the order of $10^6 \mathrm{s}^{-1}$ or $10^7 \mathrm{s}^{-1}$ for RNA folding (Pörschke, 1974), as well as a scaling factor to relate free energy differences to wall-clock time. In practice, being precise with the fastest reaction in the system is not important, as long as the fastest reaction is much faster than the remaining reactions. However, it is important to find a value that matches the scaling of free energy differences, and previous results for Kinfold simulations concluded that 10^5 is a reasonable default (Helmling *et al.*, 2017).

The option --t-fast sets the coarse graining strength by specifying a threshold what timescales should be considered instantaneous. This means, whenever the expected waiting time ($1/k_{xy}$ for a transition $m_{x \to y}$) is shorter than --t-fast, then the reaction is considered instantaneous. Internally, --t-fast is translated to an energy barrier ΔG separating conformations as: $\Delta G = -RT \cdot \ln((1/$--t-fast$)/$--k0$)$. Note that --t-fast $= 0.004$ corresponds to a time scale that is 10 times shorter than the default extension time --t-ext $= 0.04$. Unless --t-fast is explicitly specified, it is always set relative to the transcription rate as --t-ext$/10$. We do not recommend to use a --t-fast parameter that is closer (or even higher) than the --t-ext, as this would mean that the timescale of nucleotide extension is considered infinitely fast during coarse graining.

The option --o-prune sets the occupancy threshold when determining the fraction of parent structures during graph pruning (see main text). A large value, e.g., --o-prune $= 0.1$ means that up to 10% of combined occupancy are discarded after each simulation. Generally, a lower value is better, as structures with low occupancy can still become important, or at least be good parent conformations in the sense that important structures can be found in their fraying neighborhood. The default --o-prune $= 0.05$ was chosen to remove a low amount of combined occupancy, while still enabling comparatively fast simulations for RNA molecules of up to 200 nt. The parameter is also related to --k0: larger k_0 means more simulation time per nucleotide. As ensemble diversity typically decreases with simulation time (see Suppl. Sec. 2), this means there are fewer structures with more occupancy, and thus the threshold --o-prune can be lower.

The parameter --delth is a threshold for caching old candidate reactions. As discussed in main text Sec. 2.5., not every structure with low occupancy is removed right away. We cache candidate and parent conformations as well as their associated reactions for multiple transcription steps to save computation time in case the structure becomes a candidate structure again. The default --delth $= 10$ discards structures if they have not been re-identified as candidate

structures for 10 consecutive transcription steps. This parameter should have little effect on results, but larger values keep data of previously found candidate reactions for future simulations at the cost of an increased memory consumption.

2 Structure prediction of tRNAs with `DrTransformer`

Here, we are interested to see if cotranscriptional folding simulations can be used to improve structure prediction over the corresponding thermodynamic model. The latter is known to perform poorly on tRNA structures, which could – in part – be because the arms of tRNAs fold cotranscriptionally. In Suppl. Fig. 1, we see a comparison of tRNA structure prediction using `DrTransformer` and `RNAfold` (Lorenz *et al.*, 2011). 557 tRNA sequences were taken from the archive II dataset (Sloma and Mathews, 2016) and their known structure compared with (a) `RNAfold` prediction and (b) the most occupied structure at the end of a `DrTransformer` simulation. For comparison, we calculate base-pair distances between prediction and native structure, and we compare three time points: 0.04 s, 1 minute and 1 hour after transcription. Generally, the prediction quality of `DrTransformer` and `RNAfold` is similar; `DrTransformer` performs slightly worse right after transcription, but equally good after a minute or more of post-transcriptional simulation time.

We have not further investigated the differences between tRNAs that are better predicted by `DrTransformer` and tRNAs that are better predicted by `RNAfold`, but we were interested to compare the results for a range of different non-default parameters (see Table 1). As expected, the parameters may have influences on the folding of individual molecules, but there is no indication that some non-default settings are improving results in general. In conclusion, cotranscriptional folding (with the current kinetic model used by `DrTransformer`) does not yield a better tRNA structure prediction than MFE folding.

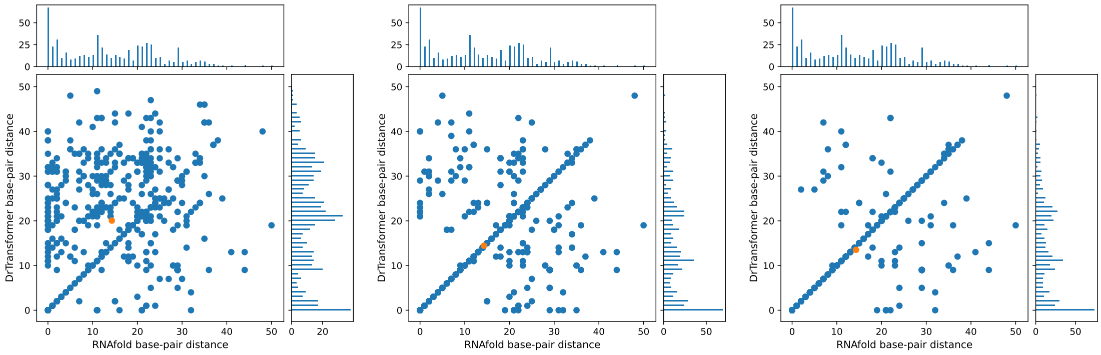

(a) Default parameters (see Tab. 1) & `--t-end` = 0.04, 60, 3600

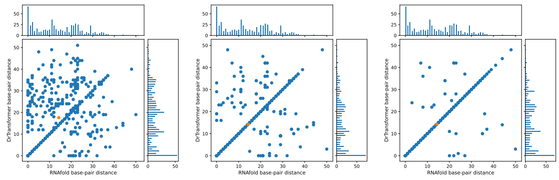

(b) `--k0` = 10^6

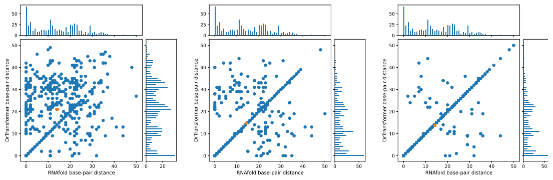

(c) `--t-ext` = 0.02; `--t-fast` = 0.002

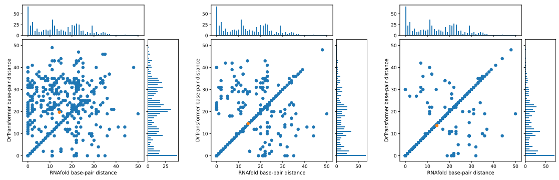

(d) `--t-fast` = $4 \cdot 10^{-4}$

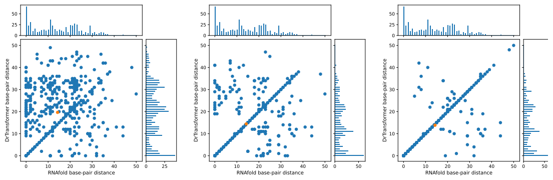

(e) `--o-prune` = 10^{-3}

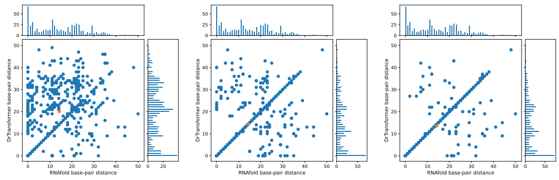

(f) `--fpwm` = 8

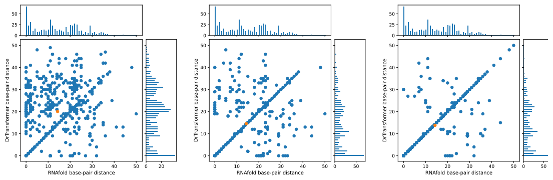

(g) `--mfree` = 12

Figure 1: tRNA structure prediction using `DrTransformer`. All plots compare the base-pair distance of `DrTransformer` and `RNAfold` to the known secondary structure. For every parameter setting we show three plots: after transcription, 1 minute after transcription, 1 hour after transcription. The orange point shows the average result over all 557 tRNA sequences taken from the archive II dataset.

3 On the diversity of cotranscriptional ensembles

The following analysis uses an artificial dataset of 380 random sequences with lengths from 20 to 200 nucleotides. All sequences were simulated with `DrTransformer` and compared to the results of 200 stochastic cotranscriptional `Kinfold` simulations. First, we show how *diverse* ensembles are at the end of transcription compared to the ensemble at thermodynamic equilibrium. Ensemble diversity is calculated as the mean base-pair distance between randomly selected structures in the ensemble

$$\mathrm{MED}(A, B) = \sum_{a \in A} \sum_{b \in B} P_a P_b d(a, b) \tag{1}$$

were P_a is the probability of structure a (in ensemble A) and $d(a, b)$ is the base-pair distance. This formula can also be used to estimate the diversity within a single ensemble ($A = B$), by assuming that structures can be chosen multiple times with the same probability.

Equilibrium ensembles of random sequences are calculated using the `ViennaRNA package` and compared to the ensembles at the end of transcription using `Kinfold` and `DrTransformer`. The results are shown in Suppl. Fig. 2. Under 60 ntlength, random sequences are predominantly at equilibrium at the end of transcription, but for longer sequences the `Kinfold` ensemble after transcription is more diverse than the equilibrium distribution. Presumably, ensemble diversity is increased because sequences have not been designed to fold into a specific metastable structure and we observe an ensemble that contains some structures that are dominant at equilibrium, as well as others which are still trapped due to the history of the transcription process.

Perhaps surprisingly, one can see that length is a good predictor for ensemble diversity at end of cotranscriptional `Kinfold` simulations. This suggests that the main source of diversity comes from small variations at the level of individual base-pairs, which are expected to increase linearly with sequence length. `DrTransformer` groups similar structures into the same δ-minimum, which means base-pair level variations are not observed. Consequently, length is not a good predictor of ensemble diversity. It appears as if the overall distribution of ensemble distances is similar for `DrTransformer` and equilibrium, but that structures with high diversity at the end of transcription can have low diversity at equilibrium and vice versa. In the direct comparison of cotranscriptional ensembles from `Kinfold` and `DrTransformer` we see that the `Kinfold` ensemble is more diverse (as expected), but we also now observe a trend where molecules with less diversity in `Kinfold` have less diversity in `DrTransformer`.

Finally, one can see that both `Kinfold` and `DrTransformer` ensembles at the end of transcription have a similar mean ensemble distance to the equilibrium ensemble. Presumably, base-pair level variations cancel when comparing `Kinfold` with equilibrium distributions, and the overall distances to dominant metastable structures dominate this analysis. This would suggest that `Kinfold` and `DrTransformer` are able to identify similar metastable structure candidates.

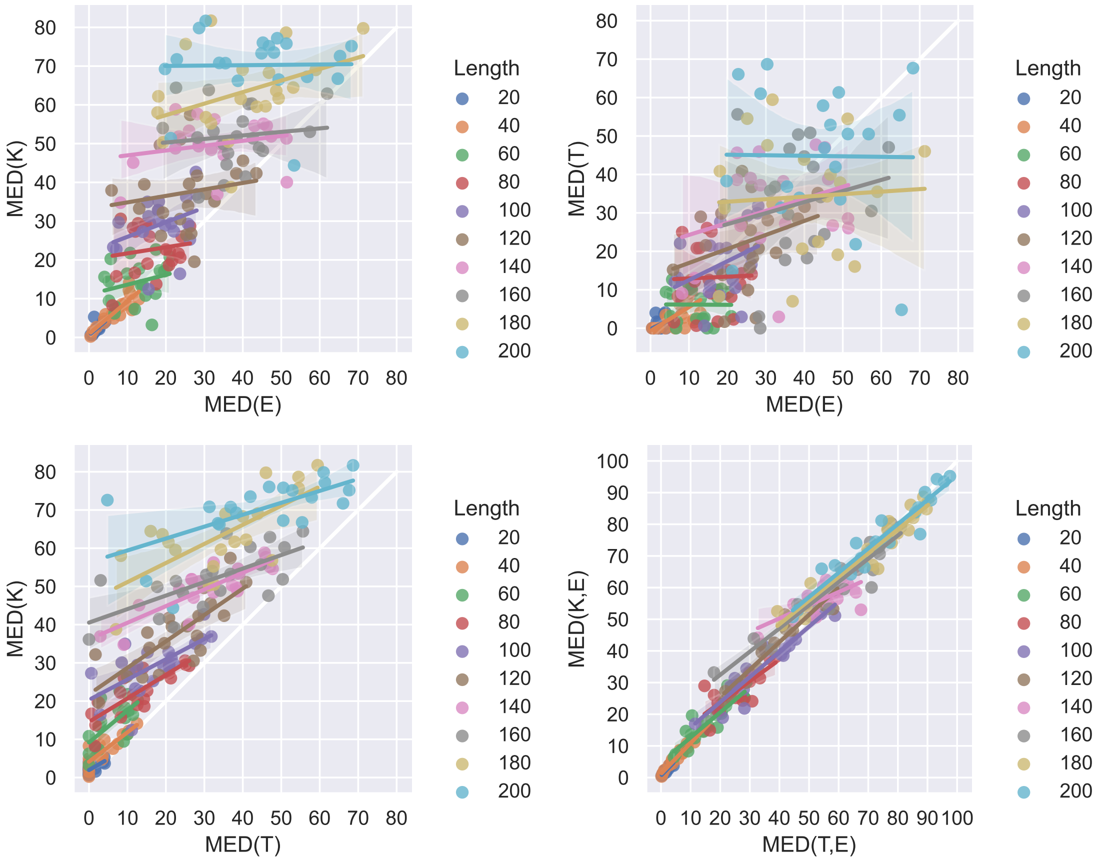

Figure 2: **Mean ensemble distances (MED).** Top left: The mean ensemble distance at the end of transcription calculated by `Kinfold` is larger than the mean ensemble distance at equilibrium. Within a specific length cohort, the ensemble distance at the end of transcription remains largely constant. Top right: The mean ensemble distance at the end of transcription calculated from `DrTransformer` simulations is distributed similar to the ensemble distance at equilibrium, but structures with high diversity at the end of transcription can have low diversity at equilibrium and vice versa. Bottom left: The mean ensemble distance at transcription end calculated by `Kinfold` and `DrTransformer` shows that `Kinfold` has a higher diversity, but it also shows that molecules with less diversity in `Kinfold` have less diversity in `DrTransformer`. Bottom right: The mean ensemble distance between `Kinfold` transcription end and equilibrium in comparison to the mean ensemble distance between `DrTransformer` transcription end and equilibrium. Both the `Kinfold` and the `DrTransformer` ensembles have similar distance to the equilibrium distribution.

4 Comparison of `Kinfold` and `DrTransformer` by varying k_0

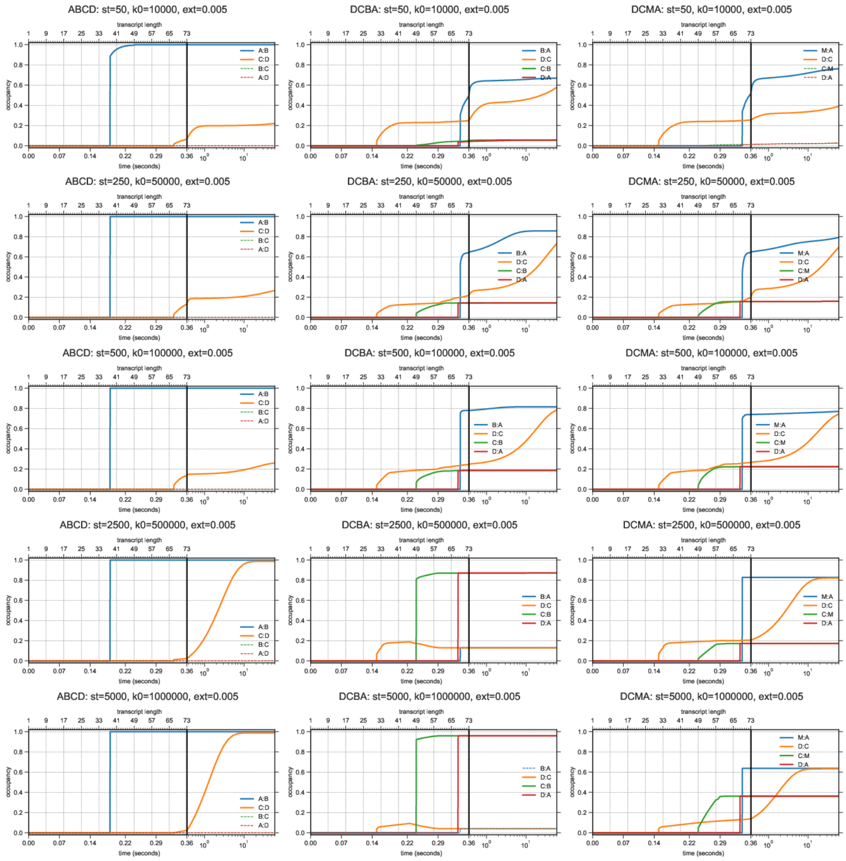

Figure 3: `DrTransformer` simulations of the three molecules 'ABCD', 'DCBA', 'DCMA' designed in Xayaphoummine *et al.* (2007) with varying simulation time per nucleotide. The extension time per nucleotides is constant at **ext**=0.005 $\mathrm{s\,nt^{-1}}$ which corresponds to a transcription rate of 200 $\mathrm{nt\,s^{-1}}$, which is unusually high, but was suggested in the original publication. k_0 is varied by two orders of magnitude: **k0**= $\{10^4, 5\cdot 10^4, 10^5, 5\cdot 10^5, 10^6\}$, where 10^5 is the `DrTransformer` default parameter. The simulation time per nucleotide in arbitrary units **st** is provided for comparison to a different transcription rate in Suppl. Fig. 5. The corresponding `Kinfold` simulations are shown in Suppl. Fig. 4. Experimental results for DCBA suggest 90% in D:A, C:B conformation and 10% in D:C, B:A conformation (Xayaphoummine *et al.*, 2007), which is in between the results for $k_0 = 5\cdot 10^5$ and $k_0 = 10^6$. Accordingly, experimental results for DCMA suggest 50% in D:A, C:M conformation and 50% in D:C, M:A conformation (Xayaphoummine *et al.*, 2007), which would also suggest that k_0 must be chosen between $k_0 = 5\cdot 10^5$ and $k_0 = 10^6$.

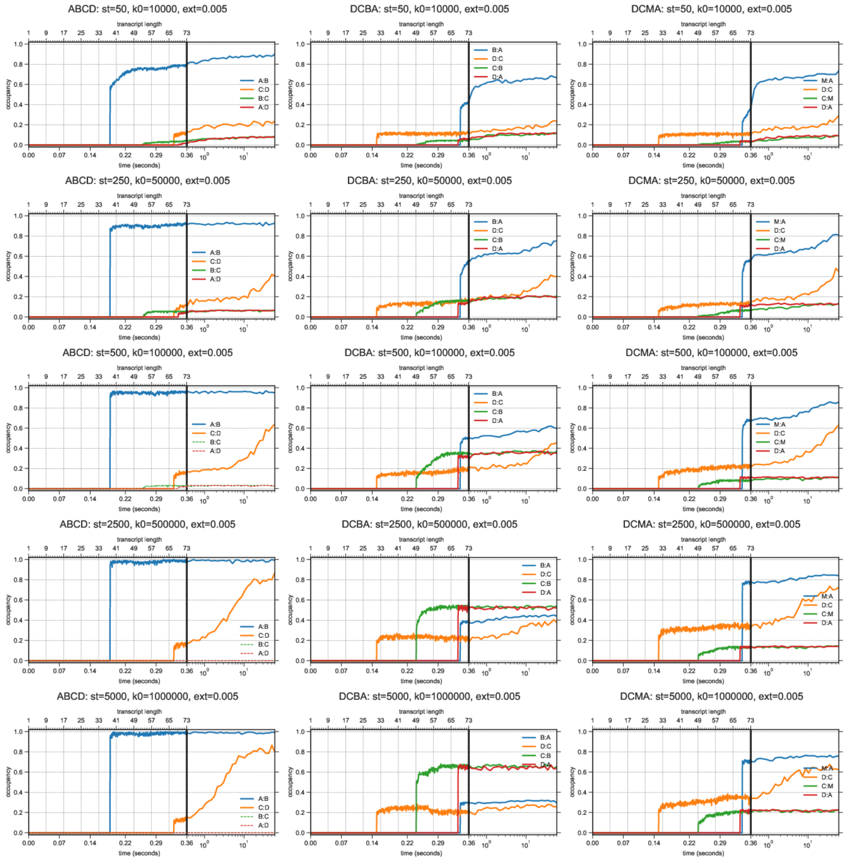

Figure 4: `Kinfold` simulations of the three molecules 'ABCD', 'DCBA', 'DCMA' designed in Xayaphoummine *et al.* (2007) with varying simulation time per nucleotide. The extension time per nucleotides is constant at **ext**=0.005 $\mathrm{s\,nt^{-1}}$ which corresponds to a transcription rate of $200\,\mathrm{nt\,s^{-1}}$, which is unusually high, but was suggested in the original publication. k_0 is varied by two orders of magnitude: $\mathbf{k0}=\{10^4, 5\cdot10^4, 10^5, 5\cdot10^5, 10^6\}$, where 10^5 is the `DrTransformer` default parameter. The simulation time per nucleotide in arbitrary units **st** is provided for comparison to a different transcription rate in Suppl. Fig. 6. The corresponding `DrTransformer` simulations are shown in Suppl. Fig. 3. Experimental results for DCBA suggest 90% in D:A, C:B conformation and 10% in D:C, B:A conformation (Xayaphoummine *et al.*, 2007), which would suggest that k_0 must be chosen to be larger than 10^6. Accordingly, experimental results for DCMA suggest 50% in D:A, C:M conformation and 50% in D:C, M:A conformation (Xayaphoummine *et al.*, 2007), which also suggests that k_0 must be chosen to be larger than 10^6.

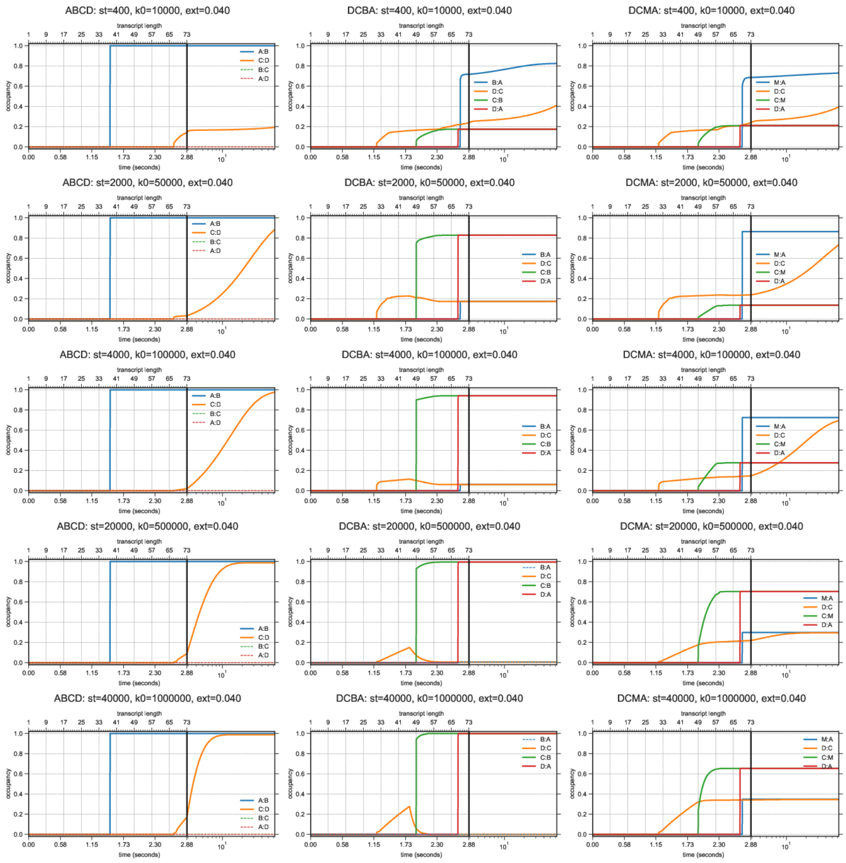

Figure 5: `DrTransformer` simulations of the three molecules 'ABCD', 'DCBA', 'DCMA' designed in Xayaphoummine *et al.* (2007) with varying simulation time per nucleotide. The extension time per nucleotides is constant at **ext**=0.040 $\mathrm{s\,nt^{-1}}$ which corresponds to a transcription rate of $25\,\mathrm{nt\,s^{-1}}$ (the `DrTransformer` default parameter). k_0 is varied by two orders of magnitude: **k0**=$\{10^4, 5\cdot 10^4, 10^5, 5\cdot 10^5, 10^6\}$, where 10^5 is the `DrTransformer` default parameter. The simulation time per nucleotide in arbitrary units **st** is provided for comparison to a different transcription rate in Suppl. Fig. 3. The corresponding `Kinfold` simulations are shown in Suppl. Fig. 6. Experimental results for DCBA suggest 90% in D:A, C:B conformation and 10% in D:C, B:A conformation (Xayaphoummine *et al.*, 2007), which would suggest that `DrTransformer` default parameters ($k_0 = 10^5$ at 25 $\mathrm{nt\,s^{-1}}$) give the best fit. Experimental results for DCMA suggest 50% in D:A, C:M conformation and 50% in D:C, M:A conformation (Xayaphoummine *et al.*, 2007), which would suggest that k_0 must be chosen between $k_0 = 10^5$ and $k_0 = 5\cdot 10^5$, however, in practice is is difficult to get this 50/50 distribution for DCMA structures due to coarse graining effects.

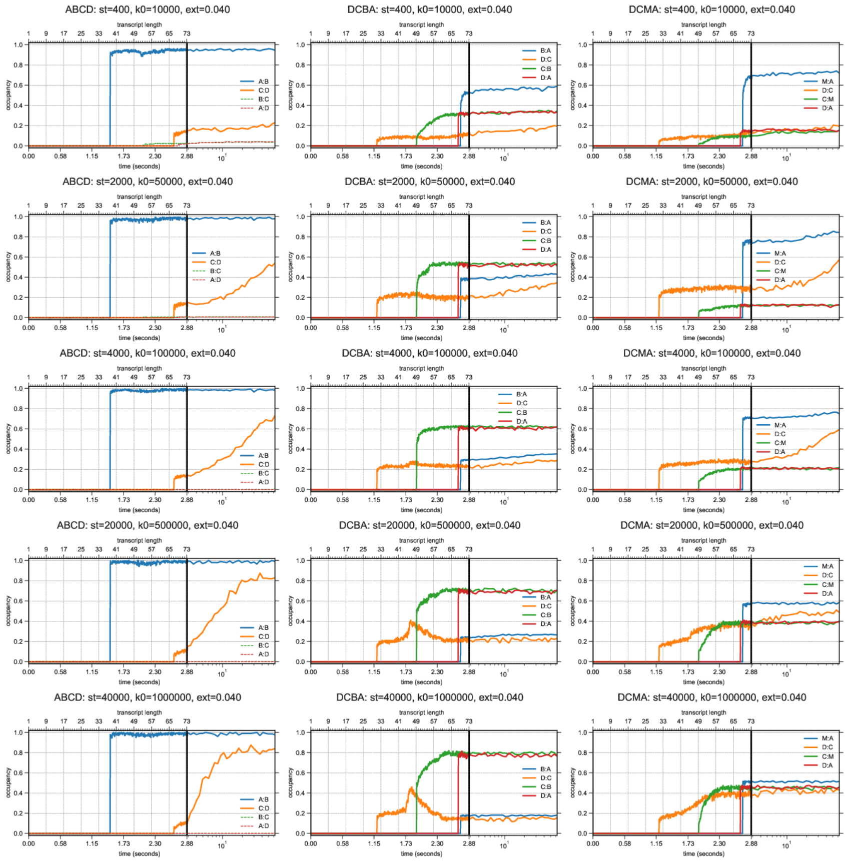

Figure 6: `Kinfold` simulations of the three molecules 'ABCD', 'DCBA', 'DCMA' designed in Xayaphoummine *et al.* (2007) with varying simulation time per nucleotide. The extension time per nucleotides is constant at **ext**=0.040 $\mathrm{s\,nt^{-1}}$ which corresponds to a transcription rate of 25 $\mathrm{nt\,s^{-1}}$ (the `DrTransformer` default parameter). k_0 is varied by two orders of magnitude: **k0**=$\{10^4, 5\cdot10^4, 10^5, 5\cdot10^5, 10^6\}$, where 10^5 is the `DrTransformer` default parameter. The simulation time per nucleotide in arbitrary units **st** is provided for comparison to a different transcription rate in Suppl. Fig. 4. The corresponding `DrTransformer` simulations are shown in Suppl. Fig. 5. Experimental results for DCBA suggest 90% in D:A, C:B conformation and 10% in D:C, B:A conformation (Xayaphoummine *et al.*, 2007), which would suggest that k_0 must be chosen to be larger than 10^6. Experimental results for DCMA suggest 50% in D:A, C:M conformation and 50% in D:C, M:A conformation (Xayaphoummine *et al.*, 2007), which is well approximated by $k_0 = 10^6$.

References

References

Bompfünewerer, A. F. *et al.* (2008). Variations on RNA folding and alignment: lessons from Benasque. *Journal of mathematical biology*, **56**(1), 129–144.

Flamm, C. *et al.* (2001). Design of multi-stable RNA molecules. *RNA*, **7**, 254–265.

Helmling, C. *et al.* (2017). NMR structural profiling of transcriptional intermediates reveals riboswitch regulation by metastable RNA conformations. *Journal of the American Chemical Society*, **139**(7), 2647–2656.

Lorenz, R. *et al.* (2011). ViennaRNA package 2.0. *Algorithms Mol Biol*, **6**, 26.

Pörschke, D. (1974). A direct measurement of the unzippering rate of a nucleic acid double helix. *Biophysical chemistry*, **2**(2), 97–101.

Sloma, M. F. and Mathews, D. H. (2016). Exact calculation of loop formation probability identifies folding motifs in RNA secondary structures. *RNA*, **22**(12), 1808–1818.

Xayaphoummine, A. *et al.* (2007). Encoding folding paths of RNA switches. *Nucleic Acids Research*, **35**(2), 614–622.
